# Biomechanical Determinants of Change of Direction Performance: A Systematic Review

**DOI:** 10.1007/s40279-025-02278-3

**Published:** 2025-07-16

**Authors:** Utkarsh Singh, Anthony S. Leicht, Jonathan D. Connor, Sara M. Brice, Adon Alves, Kenji Doma

**Affiliations:** 1 Sport and Exercise Science, College of Healthcare Sciences, James Cook University, 1 James Cook Drive, Townsville, QLD 4811 Australia; 2 Australian Institute of Tropical Health and Medicine, James Cook University, Townsville, QLD Australia; 3 Physical Sciences, College of Science and Engineering, James Cook University, Townsville, QLD Australia

## Abstract

**Background:**

The ability to change direction rapidly is crucial for enhancing performance in multidirectional sports. Evidence suggests that several biomechanical variables are associated with faster change of direction (COD) completion times. However, while it is understood that biomechanical factors influence COD performance, the evidence remains unclear because of the diverse range of biomechanical factors, inconsistent findings and potential influences from various moderating factors (e.g. sex, training experience).

**Objective:**

The primary aim of this systematic review was to identify the biomechanical determinants of COD performance while the secondary aim was to examine the impact of moderating factors on the determinants. The findings of this review could assist practitioners in designing effective training and coaching strategies to improve COD performance.

**Methods:**

A systematic literature search was conducted across the electronic databases of Scopus, PubMed, Web of Science, CINAHL, and SPORTDiscus. Studies were considered eligible if they involved healthy participants, considered biomechanical determinants of COD performance via correlational analyses and reported COD performance (i.e. time to completion). The quality of the study was assessed via the Kmet scale while study findings were collated.

**Results:**

A total of 13 studies met the inclusion criteria and analysed 45–180° COD tasks involving 374 participants. Kmet scores ranged from 73 to 96%, indicating good-to-excellent methodological quality of studies. Several biomechanical variables were identified as contributors to quicker COD completion times, including shorter ground contact time, higher approach and exit velocities, increased braking and propulsive forces, greater trunk inclination angle, lower centre-of-mass height, and increased moments and power at the hip, knee and ankle. With respect to moderating factors, included studies utilised various COD tasks (45–180°), examined mostly male participants (79.4%) with inconsistent reporting of playing/training experience and all consisted of a pre-planned COD task only.

**Conclusions:**

Our findings identified several key biomechanical variables that were important determinants of faster COD performance. However, the impact of moderating factors on COD performance was minimally examined in prior studies and requires further investigation. Recommendations are provided in this paper focussing on biomechanical contributors (e.g. ground contact time, approach velocity, braking forces), which may assist coaches with relevant training modalities to enhance COD performance.

**Supplementary Information:**

The online version contains supplementary material available at 10.1007/s40279-025-02278-3.

## Key Points

**Table:** 

Biomechanical variables including shorter ground contact times during the final foot contact for most of the change of direction tasks, higher approach and exit velocities, increased braking and propulsive forces, greater trunk inclination angle, lower centre-of-mass height, and increased moments and power at the hip, knee and ankle were identified as key contributors to quicker change of direction completion times.
A variety of pre-planned change of direction tasks (45–180°) were examined with moderating factors such as participant sex and playing/training experience rarely considered, which requires further investigation.
Implementation of specific training modalities (e.g. plyometric exercises, resistance exercises) and coaching cues were recommended to practitioners for the improvement of specific biomechanical attributes and change of direction performance.

## Introduction

The ability to rapidly change direction is considered important for successful performance in sports that involve multi-directional movements [1]. Change of direction (COD) movements are evaluated either under pre-planned conditions, where athletes follow a predetermined direction or movement pattern [2, 3], or under unplanned conditions, often referred to as agility, where athletes must react to a stimulus presented during the movement [4]. COD manoeuvres are commonly performed in sports such as soccer, rugby union and league, netball and cricket, with directional changes that ranged from 0 to 180° during competition [5–7]. Because of the importance and frequency of these manoeuvres in competition, athletes typically engage in training to meet the physical demands of multi-directional sports and develop techniques for COD ability [8]. Training aimed at enhancing COD performance (i.e. reducing completion time) primarily focuses on physical fitness factors (e.g. bioenergetics, strength, force production and neuromuscular activation) and technical factors (e.g. biomechanics) [9–11]. Specifically, it is proposed that the technical aspects of COD tasks, including biomechanical and neuromuscular components, underpin performance [1, 9].

Each COD task is a complex motor activity comprising multiplanar movements [2] with performance resulting from different biomechanical characteristics [12, 13]. Ground reaction force (GRF) characteristics, such as braking and propulsive forces, have been linked to COD performance [9]. Given that a COD task involves a linear sprint followed by a change of direction, braking forces are applied to decelerate, followed by the application of propulsive forces to reaccelerate in the new direction [14]. The application of force over a shorter duration (i.e. greater impulse) may result in better performance [15]. It is worth noting that as the time available to apply force decreases, the mean force must increase to either maintain or enhance impulse and performance (i.e. impulse = force × time). However, the force demands may vary depending on the COD angle. For example, a substantial amount of braking is required for sharper cuts (≥ 90°) compared with shallower cuts [14], and those with greater approach velocity or momentum [16]. Although understanding how forces are applied is crucial to COD performance, a comprehensive perspective must also account for whole-body movement strategies. Apart from GRF characteristics, whole-body kinematics and kinetics have been shown to influence COD performance. For instance, Marshall et al. [17] reported that maximum plantar flexor moment (*r* = 0.65) and power (*r* = 0.77), maximum thorax rotation angle (*r* = 0.51), and shorter ground contact time [GCT] (*r* = − 0.48) were significantly correlated with faster performance during a 75° COD task. For a 45° COD task, faster performance was associated with greater average sagittal hip power generation, and greater peak hip flexor and plantar flexor moments. During a 90° COD task, greater average frontal plane hip power generation and peak knee extensor moment were associated with faster 90° COD performance [13]. Further, faster performance during a modified 505 COD was associated with a greater trunk inclination angle [9, 18]. This diversity of biomechanical contributors to COD performance studies may be due to differences in the methodological design (e.g. run-up length, inclusion or exclusion of a reactivity component during the COD task, COD angle). Further, moderating factors such as athlete sex and status (i.e. elite, sub-elite) and training experience may affect these COD biomechanical contributors. For example, biomechanical characteristics were reported to differ between male and female athletes when performing the same COD task [19]. The diverse range of biomechanical variables investigated in studies examining COD performance [1], coupled with the multitude of COD tasks (i.e. COD angle range between 0° and 180°) [13] and the influence of moderating factors [19, 20], have posed challenges for researchers and practitioners to directly translate findings into their practice. As a result, prioritising key variables may prove advantageous.

While understanding that biomechanical factors influence COD performance [13, 17, 21], the coherent evidence for how these factors contribute remains unclear because of the diverse range of biomechanical factors. This lack of clarity emphasises the necessity for a more profound understanding of these contributors, taking into account moderating factors. This enhanced understanding can guide practitioners in prioritising training that aligns with the specific demands of sports. Previous narrative reviews have explored the relationship between biomechanical contributors to COD tasks and injury risk, but limited attention has been given explicitly to COD performance [12, 22]. For example, these reviews examined the association of several biomechanical factors such as lower limb joint loading with knee injury risk, while paying limited attention to the biomechanical contributors to COD performance. Identifying the biomechanical contributors to faster COD performance can help coaches design effective training practices, which are essential for success in multidirectional sports. Consequently, a systematic review can provide a comprehensive summary of biomechanical contributors to COD performance to assist practitioners in developing coaching and training strategies directly relevant to enhancing COD performance. Therefore, the primary aim of this systematic review was to identify the biomechanical determinants of COD performance while the secondary aim was to examine the influence of moderating factors on these contributors.

## Methods

This review followed most of the Preferred Reporting Items for Systematic Reviews and Meta-Analyses (PRISMA) guidelines relevant to our systematic review [23]. A review protocol was not pre-registered for this study.

### Search Strategy

Literature searches were conducted across the electronic databases of PubMed, CINAHL, SPORTDiscus, Web of Science and SCOPUS on 14 September, 2023 and updated on 5 April, 2024. The search strategy was conducted using (in different combinations) the Boolean operators AND/OR with the following keyword terms: ("COD" OR "Change of direction" OR "Agility" OR "unplanned" OR "planned" OR "anticipat*" OR "unanticipat*" OR "side*" OR “cut” OR "cutt*") AND (Biomechanic* OR Mechanic* OR kinematic* OR kinetic*) AND ("performance" OR "time-to-completion" OR "time to completion") AND (athlete OR sports OR "team sports" OR "soccer" OR "rugby" OR "AFL" OR "football" OR "cricket" OR "hockey" OR “netball” OR “basketball” OR “volleyball” OR “handball”).

### Eligibility Criteria

Studies were considered eligible and included in the systematic review upon meeting the following criteria: (1) original research studies; (2) incorporated healthy participants with no age restrictions; (3) considered biomechanical determinants of COD performance (i.e. completion time) via correlational analyses [24]; and (4) reported time to completion of COD task. Studies were excluded if: (1) they were conference presentations, posters or case studies; (2) participants were unhealthy (e.g. injuries, recent surgery); (3) they did not provide any correlational data between biomechanical characteristics and COD performance outcome; or (4) they did not examine COD.

### Selection Process

The titles, abstracts and full-text version of retrieved studies were independently screened by two researchers (US and AA). The studies were classified as meeting the inclusion criteria (yes), could be included (maybe) or not meeting the inclusion criteria (no). During the search and review process, any differences between screening authors regarding the inclusion and exclusion of studies were resolved through consensus with a third researcher (KD). The reference lists of the included articles were also examined to identify any additional relevant studies.

### Data Extraction

Following the retrieval of full-text articles, data relating to participant characteristics (e.g. age, sex, body mass, height, training experience), methodological design, aims, outcome measures and analysis (e.g. correlation between biomechanical variable and COD performance), and main findings were extracted and collated into a Microsoft Excel file (Microsoft Corporation, Redmond, WA, USA).

### Quality Assessment

The methodological quality of the included studies was evaluated using the assessment checklist proposed by Kmet et al. [25] for quantitative studies. This checklist consisted of 14 items related to study design, subject selection methods, random allocation procedures, outcome measures, sample size, estimates of variance, confounding factors, reporting of results and the conclusion obtained from all outcome measures. Items 5–7 were related to random allocation and blinding of subjects and investigators and were excluded to better align the methodological design of the included studies. The included studies were scored based on the following criteria: 2 points (yes), 1 point (partial) and 0 points (no). Items 5–7 were replaced by another three items relating to confounding factors (i.e. inclusion/exclusion of warm-up exercises, familiarisation session and playing/training experience.) [24, 26] and scored as follows: item 5 was scored a 2 if the studies had provided information about the type of warm-up exercises that were performed prior to the COD task, a 1 if studies had just mentioned that warm-up was performed and a 0 if no information regarding the warm-up was reported; item 6 was scored a 2 if the information regarding playing and training experience was reported, a 1 if either playing or training experience was reported, a 0 if no information related to training or playing experience was provided; and item 7 was scored a 2 if the studies performed a familiarisation session and had informed participants regarding recovery duration between the familiarisation session and data collection session, a score of 1 if studies had just reported that the familiarisation session was performed, and 0 if no information regarding the familiarisation was reported. Last, item 12 was excluded as it was related to study confounders, which was addressed by our replaced items (i.e. 5, 6 and 7), leaving 13 items in total [24]. As each item was scored out of 2, the maximum possible score was 26 based on 13 items. The Kmet quality score was calculated by dividing the obtained score by the maximum possible score and expressed as a percentage (e.g. a total score of 13 would be expressed as 13/26 × 100 = 50%). The Kmet quality scores of < 50%, 50–66.9%, 67–84% and > 84% were interpreted as poor, fair, good and excellent, respectively [24].

## Results

### Literature Search

**Fig. 1:**
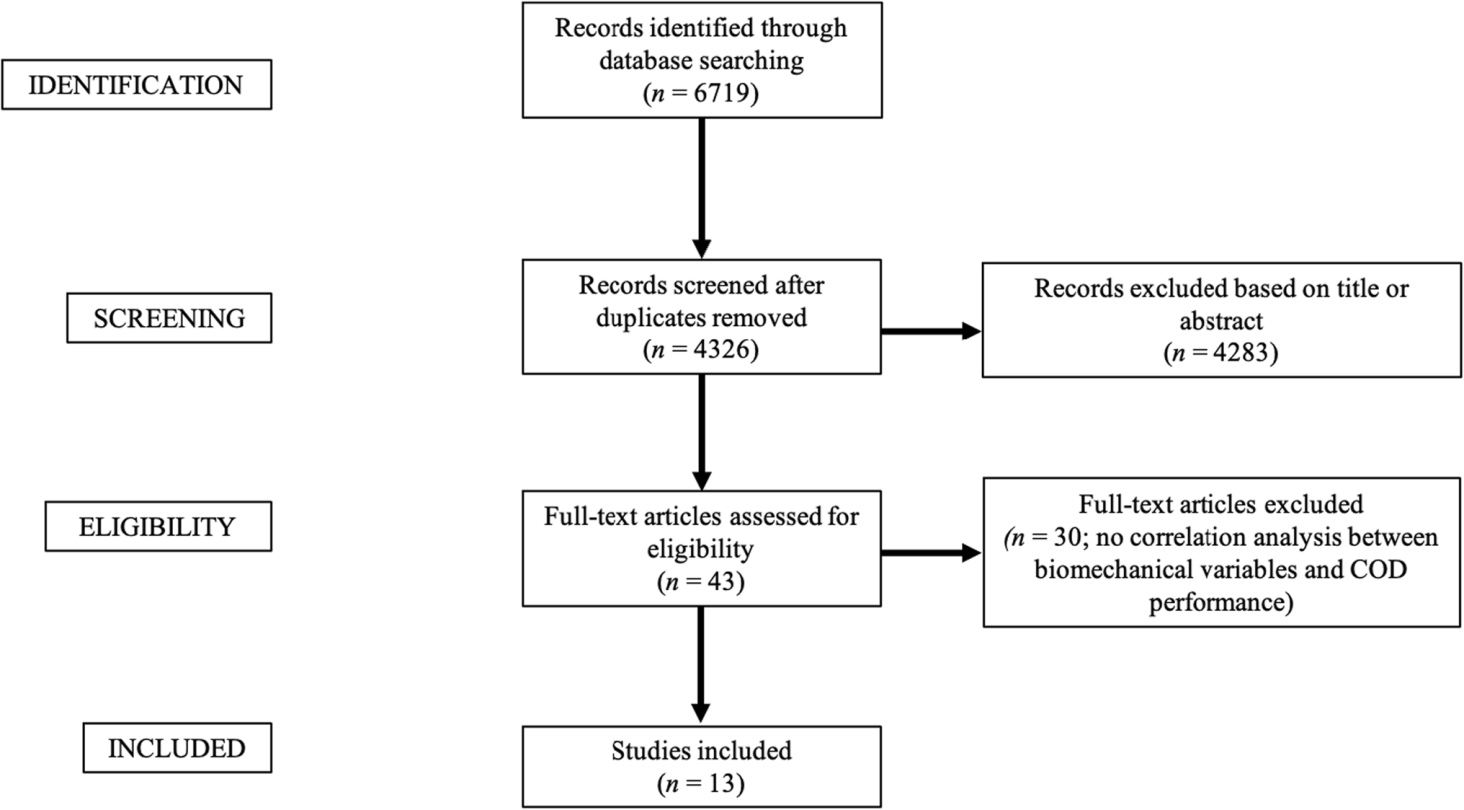
Flow chart illustrating the study selection process. *COD* change of direction

A total of 6719 abstracts were identified from the databases (Fig. 1). Following the removal of duplicates, 4326 abstracts were assessed for eligibility with the full text of 43 studies reviewed and resulting in 13 studies satisfying the inclusion criteria (Fig. 1).

### Methodological Quality

The average Kmet score of the included studies was 81.36% with five studies of excellent quality, seven studies of good quality, and one study of fair quality (Table 1). Most of the included studies addressed the following Kmet scoring items: study design, subject selection methods, random allocation procedures, outcome measures, estimates of variance, reporting of results and the conclusion obtained from all outcome measures. The least reported Kmet items were confounding factors (i.e. information regarding familiarisation session and training/playing experience) and sample size.

**Table 1:** Quality assessment of the studies using the Kmet score

Study	Item 1	Item 2	Item 3	Item 4	Item 5	Item 6	Item 7	Item 8	Item 9	Item 10	Item 11	Item 13	Item 14	Score (%)
Baena-Raya et al. [28]	2	2	2	2	2	2	2	2	1	2	2	2	2	96.15
Dos’Santos et al. [21]	2	2	2	2	2	0	0	2	1	2	2	2	2	80.77
Dos’Santos et al. [9]	2	2	2	2	2	1	0	2	2	2	2	2	2	88.46
Dos’Santos et al. [15]	2	2	2	2	2	1	0	2	2	2	2	2	2	88.46
Dos’Santos et al. [8]	2	2	2	2	2	1	0	2	2	2	2	2	2	88.46
Havens and Sigward [13]	2	2	2	2	1	1	0	2	2	2	2	2	2	84.62
Jones et al. [27]	2	2	2	2	0	1	1	2	0	2	2	2	2	76.92
Jones et al. [11]	2	2	2	2	0	0	1	2	0	2	2	2	2	73.08
Marshall et al. [17]	2	2	2	2	2	1	0	2	0	2	2	2	2	80.77
McBurnie et al. [29]	2	2	2	2	2	0	0	2	1	2	2	2	2	80.77
Sasabe et al. [18]	2	2	2	2	0	0	0	2	0	2	2	2	1	65.38
Sasaki et al. [30]	2	2	2	2	1	0	0	2	0	2	2	2	2	73.08
Welch et al. [1]	2	2	2	2	2	0	0	2	1	2	2	2	2	80.77

### Study Characteristics

There was a total of 374 participants (male, *n* = 297; female, *n* = 62; unidentified for sex, *n* = 15) within all included studies. One study examined youth (age < 18 years) athletes and the remaining 12 studies examined adults (age > 18 years) who played team sports such as soccer, rugby, basketball and cricket from collegiate to national level. Reporting of training and/or playing experience of athletes was inconsistent across studies with six [8, 9, 13, 15, 17, 27] reporting playing experience (≥ 4 years), one [28] reporting both playing (≥ 2 years) and training experience (≥ 3 years), one [21] reporting participant’s training experience of ≥ 1 years, and five not reporting training or playing experience (Table 2).

**Table 2:** Participant characteristics in the included studies

Study	Sample size	Sex	Age, height, body weight	Sport	Training/playing experience
Baena-Raya et al. [28]	30 (17 male; 13 female)	Male and female	Male: 15.47 ± 0.72 years; 181.37 ± 7.43 cm; 68.62 ± 12.30 kg Female: 15.14 ± 0.79 years; 175.64 ± 9.68 cm; 65.80 ± 10.89 kg	National level basketball players	≥ 2 years playing experience; ≥ 3 years training experience
Dos’Santos et al. [21]	40	Male	23 ± 2.9 years; 182 ± 7 cm; 88.05 ± 12.86 kg	21 sub-elite rugby league players; 19 collegiate athletes (soccer, rugby, cricket)	≥ 1 year resistance training experience
Dos’Santos et al. [9]	61	Male	20.7 ± 3.8 years, 177 ± 6.6 cm, 74.7 ± 10 kg	Soccer, rugby and cricket	≥ 5 years playing experience
Dos’Santos et al. [15]	61	Male	20.7 ± 3.8 years, 177 ± 6 cm, 74.7 ± 10.0 kg	43 soccer, 10 rugby, 7 cricket, field and 1 hockey	≥ 5 years playing experience
Dos’Santos et al. [8]	20	Male	23.8 ± 3.8 years, 179 ± 5 cm, 80.5 ± 10.9 kg	University-level soccer players	≥ 5 years playing experience
Havens and Sigward [13]	25 (13 male; 12 female)	Male and female	22.4 ± 3.9 years; 174 ± 10 cm; 70.9 ± 9.3 kg	Soccer players	16.7 ± 4.4 years soccer experience
Jones et al. [27]	18	Female	21.6 ± 4.3 years, 167 ± 7 cm, 60.3 ± 6.3 kg	Soccer players played in the top two tiers of English women’s football	≥ 5 years playing experience
Jones et al. [11]	19	Female	21.7 ± 4.3 years, 167 ± 7 cm, 60.5 ± 6.1 kg	Soccer players played in the top two tiers of English women’s football	NR
Marshall et al. [17]	15	NR	24.5 ± 2.8 years; 183.7 ± 6.7 cm; 83.5 ± 6.3 kg	Multidirectional sports	4.1 ± 1.7 years playing experience
McBurnie et al. [29]	34	Male	20 ± 3.2 years; 177 ± 6 cm; 73.5 ± 9.2 kg;	Soccer players	NR
Sasabe et al. [18]	14	Male	20.4 ± 1.1 years, 180.3 ± 4.9 cm, body 77.2 ± 5.2 kg	Collegiate basketball players (first division university team)	NR
Sasaki et al. [30]	12	Male	21.3 ± 1.0 years, 175 ± 5 cm, 67.7 ± 6.7 kg	Collegiate soccer players	NR
Welch et al. [1]	25	Male	23.5 ± 4.2 years, 183 ± 6 cm, 83 ± 6.9 kg	Gaelic football players	NR

*NR* not reported

There were a variety of COD tasks examined with varying directional angles including 45° (*n* = 3) [1, 13, 28], 70–90° (*n* = 2) [11, 29], 75° (*n* = 1) [17], 90° (*n* = 3) [15, 28], 110° (*n* = 1) [1] and 180° (*n* = 7) [8, 9, 18, 21, 27, 28, 30]. All studies reported outcomes using pre-planned COD tasks, where participants were provided with instructions regarding the direction of change prior to commencing the task. There were no studies that examined unplanned COD tasks (Table S1 of the Electronic Supplementary Material).

### Outcomes and Overall Findings

Table 3 presents a summary of the biomechanical parameters, including correlation coefficients of all parameters, as reported in the included studies. For simplification of the biomechanical determinants of COD performance, Table 3 was further refined, resulting in Table 4. In Table 4, the biomechanical parameters were only included when correlations (r) ≥ 0.4 between biomechanical parameters and completion time for COD tasks were identified [15]. These parameters were broadly grouped as: (1) kinematics: GCT, centre of mass (COM) positioning and approach and exit velocities; (2) kinetics: GRF; and (3) joints/segments: trunk, pelvis, hip, knee and ankle. The parameters were further examined at different instances of the COD task, including antepenultimate foot contact (APFC, third-to-last foot contact before making an intended direction change), penultimate foot contact (PFC, second-to-last foot contact before making an intended direction change) and final foot contact (FFC, last foot–ground contact just before executing a direction change) [8]. Additionally, measures were reported at several timepoints during FFC, including initial contact (IC), the braking phase (i.e. from IC to peak knee flexion), the push-off phase (i.e. from peak knee flexion to toe-off [TO]) and TO.

**Table 3:** Summary of all biomechanical variables and their correlation with COD completion times

Study	COD	Biomechanical variables
Baena-Raya et al. [28]	Modified 505 (180° COD), Modified T agility (90° COD) and V cut (45° COD)	Modified 505: Male athletes Kinematics: maximum velocity (*r* = − 0.74; *p* < 0.05), maximum acceleration (*r* = − 0.76; *p* < 0.05) and deceleration (*r* = 0.68; *p* < 0.05) Kinetics: maximum centripetal force (*r* = − 0.15; *p* > 0.05) Female athletes Kinematics: maximum velocity (*r* = − 0.61; *p* < 0.05), maximum acceleration (*r* = − 0.63; *p* < 0.05) and deceleration (*r* = 0.15; *p* > 0.05) Kinetics: maximum centripetal force (*r* = − 0.41; *p* < 0.05) Modified T agility: Male athletes Kinematics: maximum velocity (*r* = − 0.45; *p* > 0.05), maximum acceleration (*r* = − 0.51; *p* < 0.05) and deceleration (*r* = 0.41; *p* > 0.05) Female athletes Kinematics: maximum velocity (*r* = − 0.33; *p* > 0.05), maximum acceleration (*r* = − 0.31; *p* > 0.05) and deceleration (*r* = 0.61; *p* < 0.05) V cut: Male athletes Kinematics: maximum velocity (*r* = − 0.28; *p* > 0.05), maximum acceleration (*r* = − 0.74; *p* < 0.05) and deceleration (*r* = 0.24; *p* > 0.05) Kinetics: maximum centripetal force (*r* = − 0.39; *p* > 0.05) Female athletes Kinematics: maximum velocity (*r* = − 0.73; *p* < 0.05), maximum acceleration (*r* = − 0.64; *p* < 0.05) and deceleration (*r* = 0.45; *p* > 0.05) Kinetics: maximum centripetal force (*r* = − 0.60; *p* < 0.05)
Dos’Santos et al. [21]	Modified 505 (180° COD)	Left Kinematics: GCT at PFC (*r* = 0.3; *p* > 0.05) and FFC (*r* = 0.70; *p* < 0.01) Kinetics: vertical impact force at PFC (*r* = 0.12; *p* > 0.05), vertical impact force at FFC (*r* = 0.45; *p* < 0.01), HBF PFC (*r* = − 0.34; *p* ≤ 0.05), HBF FFC (*r* = 0.22; *p* > 0.05), vertical propulsive force at FFC (*r* = 0.05; *p* > 0.05), HPF FFC (*r* = − 0.57; *p* < 0.01), horizontal braking force ratio (FFC HBF/PFC HBF) (*r* = 0.43; *p* < 0.01) Right Kinematics: GCT at PFC (*r* = 0.08; *p* > 0.05) and FFC (*r* = 0.76; *p* < 0.01) Kinetics: vertical impact force PFC (*r* = 0.34; *p* ≤ 0.05), vertical impact force FFC (*r* = 0.56; *p* < 0.01),HBF PFC (*r* = 0.05; *p* > 0.05), HBF FFC (*r* = 0.33; *p* ≤ 0.05), VPF FFC (*r* = 0.12; *p* > 0.05), HPF FFC (*r* = − 0.61; *p* < 0.01), HBF ratio [FFC HBF/PFC HBF] (*r* = 0.13; *p* > 0.05)
Dos’Santos et al. [9]	Modified and traditional 505 (180° COD)	*Modified 505* Kinematics: GCT at FFC (*r* = 0.46; *p* < 0.001), angle of RPF (*r* = − 0.77; *p* < 0.001), angle of peak RBF (°)—PFC (*r* = − 0.53; *p* < 0.001) and FFC (*r* = − 0.46; *p* < 0.001) Joint kinematics: PFC peak hip flexion angle (*r* = 0.59; *p* < 0.001), PFC peak knee flexion angle (*r* = − 0.52; *p* < 0.001), PFC peak ankle dorsi-flexion angle (*r* = 0.44; *p* < 0.001), trunk inclination angle IC—PFC (*r* = − 0.40; *p* < 0.05) and FFC (*r* = − 0.35; *p* < 0.05), PFC trunk displacement (*r* = 0.33; *p* < 0.001), medial trunk flexion IC (*r* = − 0.32; *p* > 0.05) Kinetics: FFC HPF—peak (*r* = 0.47; *p* < 0.05) and mean (*r* = 0.42; *p* < 0.05), FFC horizontal to vertical peak (*r* = 0.77; *p* < 0.05) and mean (*r* = 0.74; *p* < 0.05) propulsive ratio, PFC horizontal to vertical peak (*r* = 0.51; *p* < 0.05) and mean (*r* = 0.60; *p* < 0.05) braking ratio *Traditional 505* Kinematics: approach time (*r* = 0.60; *p* < 0.001), approach velocity (*r* = − 0.34; *p* < 0.01), FFC touch-down velocity (*r* = − 0.38; *p* < 0.05), change FFC velocity (*r* = 0.32; *p* < 0.05), GCT at FFC (*r* = 0.39; *p* < 0.001), angle of RPF (*r* = − 0.66; *p* < 0.001), angle of peak RBF (°)—PFC (*r* = − 0.57; *p* < 0.001) and FFC (*r* = − 0.55; *p* < 0.001) Joint kinematics: PFC peak hip flexion angle (*r* = 0.47; *p* < 0.001), PFC peak knee flexion angle (*r* = − 0.45; *p* < 0.001), PFC peak ankle dorsi-flexion angle (*r* = 0.32; *p* < 0.001), trunk inclination angle IC—PFC (*r* = − 0.36; *p* < 0.05), medial trunk flexion IC (*r* = − 0.33; *p* < 0.05) Kinetics: FFC HPF—peak (*r* = 0.53; *p* < 0.05) and mean (*r* = 0.55; *p* < 0.05), FFC mean HBF (*r* = 0.49; *p* < 0.001), mean RPF (*r* = -0.35; *p* < 0.001), FFC horizontal to vertical peak (*r* = 0.66; *p* < 0.05) and mean (*r* = 0.68; *p* < 0.05) propulsive ratio, PFC horizontal to vertical peak (*r* = 0.52; *p* < 0.05) and mean (*r* = 0.80; *p* < 0.05) braking ratio
Dos’Santos et al. [15]	90° COD	Kinematics: PFC (*r* = − 0.660; *p* < 0.001), FFC touch-down (*r* = − 0.752; *p* < 0.001), and exit (*r* = − 0.733; *p* < 0.001) velocity; shorter approach times (*r* = 0.620; *p* < 0.001), shorter PFC and FFC GCTs (*r* = 0.551–0.581; *p* < 0.001) Kinetics: greater peak (*r* = − 0.641; *p* < 0.001) and mean RPF (*r* = − 0.530; *p* < 0.001) and ML propulsive forces (*r* = − 0.588 to − 0.627; *p* < 0.001); greater mean HPF (*r* = 0.608; *p* < 0.001), and greater PFC (*r* = 0.551; *p* < 0.001) and FFC mean HBF (*r* = 0.535; *p* < 0.001); greater mean FFC RBF (*r* = − 0.484; *p* < 0.001), greater peak VPF (*r* = − 0.449; *p* < 0.001) and HPF (*r* = − 0.460;* p* < 0.001) Joint kinematics: Lower hip flexion range of motion (*r* = 0.406; *p* < 0.001), greater initial foot progression angles (*r* = − 0.411; *p* < 0.001) Joint kinetics: greater peak KIRMs (*r* = − 0.54; *p* < 0.001), greater peak KAMs (*r* = − 0.41; *p* < 0.001)
Dos’Santos et al. [8]	Traditional 505 (180° COD)	APFC Kinematics: GCT (*r* = 0.46; *p* < 0.05), angle of peak RBF (*r* = 0.74 *p* < 0.001) Kinetics: mean vertical GRF (*r* = − 0.55; *p* < 0.05), peak VBF (*r* = − 0.47; *p* < 0.05), vertical total impulse (*r* = 0.04; *p* > 0.05), peak HBF (*r* = − 0.63; *p* < 0.05), mean horizontal GRF (*r* = − 0.74; *p* < 0.001), horizontal total impulse (*r* = − 0.53; *p* < 0.05), peak RBF (*r* = − 0.52; *p* < 0.05), mean RGRF (*r* = − 0.64; *p* < 0.001), resultant total impulse (*r* = 0.13; *p* > 0.05), peak H-to-VBF ratio (*r* = − 0.74; *p* < 0.001), mean H-to-VGRF ratio (*r* = − 0.78; *p* < 0.001) PFC Kinematics: GCT (*r* = 0.08; *p* > 0.05), angle of peak RBF (*r* = 0.48 *p* < 0.001) Kinetics: mean vertical GRF (*r* = 0.09; *p* > 0.05), peak VBF (*r* = 0.14; *p* > 0.05), vertical total impulse (*r* = 0.04; *p* > 0.05), peak HBF (*r* = 0.21; *p* > 0.05), mean horizontal GRF (*r* = − 0.30; *p* > 0.05), horizontal total impulse (*r* = − 0.34; *p* > 0.05), peak RBF (*r* = 0.33; *p* > 0.05), mean RGRF (*r* = − 0.03; *p* > 0.05), resultant total impulse (*r* = − 0.09; *p* > 0.05), peak H-to-VBF ratio (*r* = − 0.49; *p* < 0.05), mean H-t- VGRF ratio (*r* = − 0.57; *p* < 0.001) FFC Kinematics: GCT (*r* = 0.35; *p* > 0.05), angle of peak RBF (*r* = 0.36; *p* > 0.05) Kinetics: mean vertical GRF (*r* = − 0.14; *p* > 0.05), peak VBF (*r* = 0.17; *p* > 0.05), vertical total impulse (*r* = 0.27; *p* > 0.05), peak HBF (*r* = 0.01; *p* > 0.05), mean horizontal GRF (*r* = − 0.62; *p* < 0.001), horizontal total impulse (*r* = − 0.05; *p* > 0.05), peak RBF (*r* = 0.12; *p* > 0.05), mean RGRF (*r* = − 0.39; *p* > 0.05), resultant total impulse (*r* = 0.17; *p* > 0.05), peak H-to-VBF ratio (*r* = − 0.35; *p* > 0.05), mean H-to-VGRF ratio (*r* = − 0.79; *p* < 0.001)
Havens and Sigward [13]	45°and 90°	Cut 45 Kinematics: ML COM–COP separation (*r* = − 0.39; *p* > 0.05) Joint kinetics: hip extensor moment (*r* = 0.40; *p* > 0.05), hip sagittal power (*r* = − 0.48; *p* < 0.05), ankle plantar flexor moment (*r* = 0.45; *p* < 0.05) Cut 90 Kinetics: ML-GRI (*r* = − 0.49; *p* < 0.05) Joint kinematics: hip rotation angle (*r* = − 0.47; *p* < 0.05) Joint kinetics: hip frontal power (*r* = − 0.59; *p* < 0.05), knee extensor moment (*r* = 0.50; *p* < 0.05)
Jones et al. [27]	Traditional 505 (180° COD)	Kinematics: approach velocity at the start of PFC (*r* = − 0.49)
Jones et al. [11]	70–90° COD	Kinematics: velocity at start of PFC (*r* = − 0.85; *p* < 0.05), velocity at end of PFC (*r* = − 0.85; *p* < 0.05), velocity at start of FFC (*r* = − 0.84; *p* < 0.05), velocity at end of FFC (*r* = − 0.87; *p* < 0.05), minimum horizontal COM velocity during the manoeuvre (*r* = − 0.86; *p* < 0.05)
Marshall et al. [17]	75° COD	Kinematics: GCT (*r* = − 0.48; *p* < 0.05) Joint kinematics: pelvis lateral tilt range [from initial contact to peak knee flexion] (*r* = − 0.54; *p* < 0.05), maximum thorax lateral rotation (*r* = 0.51; *p* < 0.05) Joint kinetics: peak ankle power (*r* = 0.77; *p* < 0.05), peak plantar flexor moment (*r* = 0.65; *p* < 0.05)
McBurnie et al. [29]	70–90° COD	Kinematics: horizontal approach velocity at the start of PFC (*r* = − 0.58; *p* < 0.05), horizontal exit velocity (*r* = − 0.45; *p* < 0.05) Joint kinetics: peak knee abduction moment (r = − 0.59; *p* < 0.05), peak knee rotation moment (r = 0.53; *p* < 0.05), peak knee flexion moment (*r* = − 0.51; *p* < 0.05), peak hip flexor moment FFC (*r* = 0.42; *p* < 0.05)
Sasabe et al. [18]	Modified 505 (180° COD)	Kinematics: COM height (*r* = 0.54; *p* < 0.05), trunk lean angle (*r* = − 0.74; *p* < 0.05)
Sasaki et al. [30]	Modified 505 (180° COD)	Joint kinematics: trunk forward angular displacement between foot contact and maximum inclination of the trunk (*r* = 0.61; *p* < 0.05); forward inclination angle at: foot contact (*r* = − 0.10; *p* > 0.05), maximum inclination (*r* = 0.39; *p* > 0.05) and foot off (*r* = 0.04; *p* > 0.05); lateral inclination angle at: foot contact (*r* = − 0.26; *p* > 0.05), maximum inclination (*r* = − 0.50; *p* > 0.05) and foot off (*r* = 0.07; *p* > 0.05)
Welch et al. [1]	45° and 110° COD	*45° Cut* Change from the start to the end of the eccentric phase Joint kinematics: ankle rotation angles (*r* = − 0.41), ankle abduction angles (*r* = 0.40), hip abduction angles (*r* = 0.36), knee rotation angles (*r* = − 0.41) At the end of eccentric phase Joint kinematics: thorax to pelvis rotation angles (*r* = 0.36), ankle rotation angles (*r* = − 0.33) Change from the start to the end of the concentric phase Kinematics: posterior COM to knee distance (*r* = 0.35), GCT (*r* = 0.34) Joint kinematics: Ankle abduction angles (*r* = − 0.37) At toe off Kinematics: vertical COM to ankle distance (*r* = 0.37) 110° Cut Change from the start to the end of the eccentric phase: hip flexion angles (*r* = 0.59), lateral COM to knee orientation (*r* = − 0.56) End of eccentric phase Kinematics: lateral COM to ankle orientation (*r* = − 0.65), lateral COM to knee orientation (*r* = − 0.63) Joint kinematics: hip flexion angles (r = 0.54), thorax to pelvis flexion angles (r = 0.53) Change from the start to the end of the concentric phase Kinematics: time (*r* = 0.58) At toe off Kinematics: posterior COM to ankle orientation (*r* = 0.62), GCT (*r* = 0.60) Joint kinematics: pelvis abduction angles (*r* = − 0.66)

*APFC* antepenultimate foot contact, *COD* change of direction, *COM* centre of mass, *COP* center of pressure, *FFC* final foot contact, *GCT* ground contact time, *GRF* ground reaction forces, *GRI* ground reaction impulse, *H* horizontal, *HBF* horizontal braking force, *HPF* horizontal propulsive force, *IC* initial contact, *ML* medio-lateral, *PFC* penultimate foot contact, *RBF* resultant braking force, *ROM* range of motion, *RPF* resultant propulsive force, *VBF* vertical braking force, *VPF* vertical propulsive force

**Table 4:** Biomechanical variables reported across the included studies

	COD type (phase)	Description (faster/slower COD completion time)
*Kinematics*		
GCT	75° (during FFC), 90° (during PFC and FFC), 110° (during FFC), 180° modified (during FFC) and traditional 505 COD (during APFC)	Smaller = faster (*r* = 0.46–0.76)
COM to ankle distance	110° (FFC: at TO)	Smaller = faster (*r* = 0.62) [maintaining lower COM]
COM height	180° modified 505 COD (during PFC)	Smaller = faster (*r* = 0.54)
Lateral distance between knee/ankle and COM	110° (FFC: end of eccentric phase)	Greater = faster (*r* = − 0.56 to −﻿ 0.65)
Velocity	45° (maximum velocity), 70–90° (PFC: at IC, FFC: at TO), 90° (PFC: at IC, FFC: at IC and TO, maximum velocity), and traditional 180° COD (PFC: at IC, maximum velocity)	Greater = faster (*r* = − 0.45 to − 0.87)
Acceleration/deceleration	45°, 90° and 180° (maximal acceleration and deceleration)	Greater = faster (*r* = − 0.41 to − 0.76)
*Kinetics*		
Mean and peak propulsive forces	90° (during FFC), 180° modified 505 (during FFC), 180° traditional 505 COD (during FFC)	Greater = faster (*r* = − 0.42 to − 0.64)
Mean and peak braking forces	90° (during FFC), 180° traditional 505 COD (during APFC and FFC)	Greater = faster (*r* = − 0.48 to − 0.62)
Mean GRF	180° traditional 505 COD (during APFC and FFC)	Greater = faster (*r* = − 0.56 to − 0.74)
Peak vertical impact forces	180° modified 505 (during FFC)	Smaller = faster (*r* = 0.45–0.56)
Horizontal total impulse	180° traditional 505 COD (during APFC)	Greater = faster (*r* = − 0.53)
ML-GRI	90° (FFC: IC to peak knee flexion)	Greater = faster (*r* = − 0.49)
Horizontal braking forces ratio	180° modified 505	Smaller = faster (*r* = 0.43)[(indicates a greater proportion of braking force during the PFC]
Peak and mean horizontal-to-vertical braking force ratio	180° modified 505 (during PFC), 180° traditional 505 COD (during APFC and PFC)	Greater = faster (*r* = 0.49–0.80) [greater horizontal force contribution]
Peak and mean horizontal-to-vertical propulsive ratio	180° modified 505 (during FFC), 180° traditional 505 COD (during FFC)	Greater = faster (*r* = 0.66–0.77) (greater horizontal force contribution)
Mean horizontal-to-vertical GRF ratio	180° traditional 505 COD (during APFC, PFC and FFC)	Greater = faster (*r* = − 0.57 to − 0.79) [greater horizontal force contribution]
Maximum centripetal force	45° and 180°	Greater = faster (*r* = − 0.41 to −﻿ 0.60)
*Trunk and pelvis biomechanics*		
Thorax to pelvis rotation/thorax rotation angle	75° (FFC: IC to peak knee flexion)	Greater = faster (*r* = 0.51) [greater thorax rotation towards the direction of cut]
Thorax to pelvis flexion	110° (FFC: end of eccentric phase)	Smaller = faster (*r* = 0.53)
Pelvis lateral tilt range	75° (FFC: IC to peak knee flexion)	Smaller = faster (*r* = − 0.54) [smaller pelvis contralateral drop]
Pelvis abduction angles	110° (FFC: at TO)	Greater = faster (*r* = − 0.66) [lean in the direction of cut]
Trunk inclination angle	180° modified 505 (PFC: at IC)	Greater = faster (*r* = − 0.40 to − 0.74) [greater forward inclination]
*Hip biomechanics*		
Hip sagittal power generation	45° (FFC: IC to peak knee flexion)	Greater = faster (*r* = − 0.48)
Hip frontal power generation	90° (FFC: IC to peak knee flexion)	Greater = faster (*r* = − 0.59)
Peak hip flexor/extensor moment	45° (FFC: IC to peak knee flexion), 70–90° (during FFC)	Greater = faster (*r* = 0.40–0.42)
Hip rotation angle	90° (FFC: at IC)	Greater = faster (*r* = − 0.47) [greater internal rotation]
Hip flexion range of motion	90° (during FFC), 110° (start to the end of the eccentric phase)	Smaller = faster (*r* = 0.40–0.59)
Peak hip flexion angle	110° (end of the eccentric phase), 180° modified 505 (during PFC), 180° traditional 505 (during PFC)	Greater = faster (*r* = 0.47–0.59)
*Knee biomechanics*		
Peak knee flexion angle	180° modified 505 (during PFC), 180° traditional 505 (during PFC)	Greater = faster (*r* = -0.45 to − 0.52)
Peak knee abduction moment	70–90° (FFC: IC to peak knee flexion), 90° (FFC: IC to peak knee flexion)	Greater = faster (*r* = − 0.4 to − 0.59)
Peak knee rotation moment	70–90° (FFC: IC to peak knee flexion), 90° (FFC: IC to peak knee flexion)	Greater = faster (*r* = 0.53–0.54)
Peak knee flexor/extensor moment	70–90° (FFC: IC to peak knee flexion), 90° (FFC: IC to peak knee flexion)	Greater = faster (*r* = − 0.51 to − 0.50)
*Ankle and foot biomechanics*		
Peak ankle dorsi-flexion angle	180° modified 505 (during PFC)	Greater = faster (*r* = 0.43)
Ankle rotation angles	45° (FFC: during eccentric phase)	Smaller = faster (*r* = − 0.41) [less external rotation change]
Ankle abduction angles	45° (FFC: during eccentric phase)	Greater = faster (*r* = 0.40) [greater abduction change]
Initial foot progression angles	90° (FFC: at IC)	Greater = faster (*r* = − 0.41)
Peak plantar flexor moments	45° (FFC: IC to peak knee flexion), 75° (FFC: IC to peak knee flexion)	Greater = faster (*r* = 0.45–0.65)
Mean ankle power	75° (FFC: IC to peak knee flexion)	Greater = faster (*r* = 0.77)

*APFC* antepenultimate foot contact, *COD* change of direction, *COM* centre of mass, *FFC* final foot contact, *GCT* ground contact time, *GRF* ground reaction force, *GRI* ground reaction impulse, *IC* initial contact, *ML* medio-lateral, *PFC* penultimate foot contact, *TO* toe-off

#### Kinematics

Smaller GCT was associated with quicker completion times for the 75° (during FFC), 90° (during PFC and FFC), 110° (during FFC), and 180° traditional (during APFC and FFC) and 180° modified 505 (during FFC) COD tasks [1, 8, 9, 15, 17, 21]. Maintenance of a low COM was linked to quicker completion times for the 110° (FFC: at TO), and 180° modified 505 (during PFC) COD tasks [1, 18]. Furthermore, quicker completion times were associated with higher approach, touch down and exit velocity during the 70–90° (PFC: at IC, FFC: at TO), 90° (PFC: at IC, FFC: at IC and TO), and 180° traditional 505 (PFC: at IC) COD tasks [15, 27, 29]. Additionally, maximum velocity, acceleration and deceleration was linked with quicker completion times during 45°, 90° and 180° COD tasks [28]. Velocity measures were not reported for the 75° COD tasks [17].

#### Kinetics

The GRF-related measures were only reported for 90° and 180° COD tasks [8, 9, 13, 15, 21] Greater mean and peak propulsive forces were associated with quicker completion times during the 90° (during FFC), 180° modified 505 (during FFC) and 180° traditional 505 (during FFC) COD tasks [9, 15, 21]. Greater peak and mean braking forces were linked with quicker completion times during the 90° (during FFC), and 180° traditional 505 (during APFC and FFC) COD tasks [8, 15]. Furthermore, greater mean GRF was associated with faster completion times during the 180° traditional 505 (during PFC and FFC) COD tasks [8]. A larger horizontal-to-vertical braking force ratio and a greater horizontal-to-vertical propulsive force ratio was linked to quicker completion times during the 180° modified and traditional 505 COD tasks [8, 9]. Additionally, a larger horizontal-to-vertical GRF ratio was linked to quicker completion times during the 180° traditional 505 (at APFC, PFC, FFC) COD task [8]. Moreover, greater horizontal and medio-lateral impulse was associated with faster completion times during the 180° traditional 505 COD test (during APFC) [8] and the 90° COD test (FFC: from IC to peak knee flexion) [13], respectively.

#### Joints/Segments

##### Trunk and Pelvis

Greater trunk rotation towards the direction of the cut (i.e. COD) was associated with faster completion times for the 75° (at FFC: IC to peak knee flexion) COD task [17]. Greater sagittal plane trunk inclination angle was linked to quicker completion times for the 180° modified 505 (PFC: at IC) COD task [9, 18].

The pelvis biomechanical measures were reported for 75° and 110° tasks only [1, 17] with less pelvis lateral tilt linked to quicker completion times for the 75° (FFC: from IC to peak knee flexion) COD task [17]. Furthermore, greater pelvis abduction was associated with quicker completion times for the 110° COD task (FFC: at TO) [1].

##### Hip

Hip biomechanical measures were reported for 45°, 70–90°, 90°, 110° and 180° COD tasks [1, 9, 13, 15, 29]. Greater hip internal rotation was associated with quicker completion times for the 90° (FFC: at IC) COD task [13]. Moreover greater hip flexion was associated with quicker completion times for the 110° (FFC: at end of the eccentric phase), while greater peak hip flexion was associated with 180° modified (during PFC), and traditional (during PFC) 505 COD tasks [1, 9]. Additionally, less hip flexion was linked to quicker completion times for the 90° (during FFC) and 110° (FFC: IC to end of the eccentric phase) COD tasks [1, 15].

Greater net hip sagittal and frontal plane power generation was associated with faster completion times for the 45° (FFC: IC to peak knee flexion) and 90° (FFC: IC to peak knee flexion) COD tasks, respectively [13]. Furthermore, a greater peak hip sagittal moment was linked to quicker completion times for the 45° (FFC: IC to peak knee flexion) and 70–90° (during FFC) COD tasks [13, 29].

##### Knee

Knee biomechanical measures were reported for 45°, 70–90°, 90° and 180° COD tasks [1, 9, 15, 29]. Greater peak knee flexion angles were associated with quicker 180° modified (during PFC) and traditional (during PFC) 505 COD completion times [9]. Further, greater peak knee flexion, abduction and rotation moments were associated with quicker completion times for both the 70–90° (FFC: IC to peak knee flexion) and 90° (FFC: IC to peak knee flexion) COD tasks [15, 29].

##### Ankle and Foot

Ankle biomechanical measures were reported for 45°, 90° and 180° COD tasks [1, 9, 13, 15, 17]. Greater peak ankle dorsiflexion was associated with faster completion times during modified 180° (during PFC) 505 COD tasks [9]. Smaller ankle transverse rotation angles and greater initial foot progression angles (i.e. angle formed by the longitudinal axis of the foot and the direction of motion) were associated with quicker 45° (FFC: during eccentric phase) and 90° (FC: at IC) COD completion times, respectively [1, 15]. Additionally, greater peak plantar flexor moments were associated with quicker 45° (FFC: IC to peak knee flexion) and 75° (FFC: IC to peak knee flexion) COD completion times [13, 17]. Greater mean net ankle power was associated with quicker 75° (FFC: IC to peak knee flexion) COD completion times [17].

## Discussion

The aim of this systematic review was to identify the biomechanical determinants of COD performance and the influence of moderating factors. Based on the inclusion criteria, 13 studies with good-to-excellent quality ratings involving 374 participants were included. Several biomechanical parameters including shorter GCT, higher approach and exit velocities, increased braking and propulsive forces, greater trunk inclination angle, lower COM height, and increased moments and power at the hip, knee and ankle were found to be associated with quicker COD completion times. With respect to moderating factors (i.e. task, sex and playing/training experience), all included studies involved pre-planned COD tasks only, and mostly (79.4%) male participants with inconsistent reporting of playing/training experience. Our study’s findings suggest that several biomechanical variables contribute to quicker COD completion times but the influence of moderating factors on these contributors is unclear. Coaches and practitioners can utilise these identified biomechanical predictors to design relevant coaching and training practices aimed at enhancing COD performance.

Based on the studies reviewed, shorter GCTs during various phases were consistently associated with quicker completion times for COD tasks (Table 4). The reduction in GCT resulted from athletes spending less time in braking and propulsive phases [31], ultimately decreasing the duration of the COD manoeuvres [21, 31]. While a shorter GCT during braking and propulsive phases is imperative for quicker completion times, we also found that greater approach and exit velocities were also linked to quicker COD completion times [15]. Therefore, maintaining and minimising velocity losses during a COD task seemed to be important for faster performance [12]. The magnitude of velocity (i.e. approach and exit) was COD angle dependent, with sharper COD tasks resulting in reduced approach and exit velocity [32, 33]. For example, Havens and Sigward [13] reported lower velocity at IC of the PFC and FFC during a 90° cut compared with a 45° cut. These findings suggested that sharper cuts (> 60°) required substantial braking, while velocity maintenance was key throughout the shallower (≤ 45°) COD tasks [34]. The reduced approach velocity noted during sharper COD tasks was likely attributable to increased braking forces during the PFC and FFC to reduce velocity and execute the intended COD task [12]. Furthermore, because a shorter GCT is ideal for faster performance [15, 17], achievement of this requires the application of greater braking forces (i.e. increased relative impulse) for deceleration, followed by greater propulsive forces to enhance impulse and maximise exit velocity, in accordance with the impulse–momentum relationship [15]. Additionally, compared with shallower COD tasks, sharper COD manoeuvres result in increased GCT due to the greater braking and propulsive force demands, as well as the higher impulse required to change momentum [32, 35]. Consequently, the effective application of both braking and propulsive forces becomes particularly crucial during sharp COD tasks to achieve quicker completion times.

Braking forces play a crucial role in reducing (i.e. deceleration) COM velocity and facilitating the rapid application of force in the new intended direction [14, 15]. Increased deceleration facilitates more rapid decreases in whole-body momentum, allowing athletes to slow down over shorter distances and/or in less time [14]. Consequently, athletes with greater deceleration capabilities can approach at higher velocities by producing greater braking forces within a shorter duration (i.e. horizontal braking impulse). This ability minimises the distance required for deceleration before executing COD, ultimately leading to faster overall COD performance times [14]. Therefore, athletes need to be adequately conditioned to generate and control high braking forces in addition to propulsive forces [15]. Further, given athletes reduce their momentum (i.e. velocity) while performing sharper COD tasks (≥ 90°) [13, 32], a PFC dominant braking strategy may be indicative of more effective deceleration (i.e. reduction in velocity) and transition from braking to push-off during a COD task [15]. It is worth noting that the choice of braking strategy (i.e. PFC dominant or APFC dominant) employed by athletes will also depend upon the approach distances within the COD task. For instance, during the modified 505 COD with a 5-m approach distance, the PFC may play a more significant role in facilitating braking compared with that during the traditional 505 COD task, which has a 15-m approach distance. During the traditional 505 task, higher approach speeds are achieved and a greater deceleration is required that may necessitate greater dependence of braking forces from earlier foot contacts [8], such as the APFC [8]. While maximum force generation is a crucial factor for COD performance, the ability to direct this force optimally offers distinct advantages for faster COD performance [10]. For example, a greater horizontal-to-vertical braking force and horizontal-to-vertical propulsive force ratios were linked to quicker 180° COD completion times in the included studies, reinforcing the contribution of horizontal forces to braking and propulsion [8, 9]. A more horizontally oriented force vector would facilitate more effective braking and net deceleration (i.e. a reduction in velocity) [10]. Additionally, a greater horizontally oriented force vector during propulsion facilitates a greater increase in velocity during push-off [36].

Because of the multi-articular nature of COD tasks, body segments (i.e. trunk, pelvis) and joints (i.e. hip, knee and ankle) play a significant role in producing a greater performance [29, 37]. For example, the current review identified that greater trunk inclination and a lower COM height were associated with quicker completion times for 180° COD tasks. Greater trunk inclination towards the desired exit direction of movement would play a key role in lowering the athlete’s COM height, as recommended by Dos’Santos et al. [9]. Lowering the COM height would also increase stability and provide athletes with an optimal position for braking and push-off [18, 38] and facilitate better re-acceleration in the desired direction for better COD performance [9, 18]. In addition to the greater trunk inclination angle, lowering the COM during 180° COD tasks can be accompanied by flexion of the hip and knee, and dorsiflexion at the ankle [9]. The current review also confirmed that greater peak sagittal hip, knee and ankle moments were associated with quicker COD completion times. The greater joint moments down the lower limb kinetic chain were likely a result of higher approach velocities that require greater braking forces [29]. This increased velocity (i.e. increased momentum) and forces would likely enhance activation of the hip extensors, potentially facilitating more controlled deceleration [29]. Simultaneously, the knee extensor muscles would act eccentrically to reduce momentum, enabling a rapid transition from braking to reacceleration in the new intended direction, ultimately improving exit velocity [13, 29]. These findings indicate the importance of strength across the lower limb joints for COD performance.

While greater joint moments are paramount for COD performance, joint power, defined as the product of joint moments and angular velocity, is also important [39]. Indeed, greater power generation at the ankle joint, coupled with larger moments and shorter GCT, have been linked to quicker completion times during a 75° COD task [17], highlighting the importance of large and fast force production. The ankle plays a crucial role in generating horizontal velocity, particularly when the COM is positioned in front of the centre of pressure [40]. Ankle power and force generation become pivotal during the late stages, especially when the COM precedes the planted foot for the new direction. Apart from power generation at the ankle joint, quicker completion time was linked with greater power at the hip joint. Hip extensors have been found to play a key role in generating power during deceleration but this may change for a 90° COD task because of greater redirectional demands [13]. When performing 90° COD tasks, hip power generation in the frontal plane, rather than the sagittal plane, predicted quicker completion times [13]. In summary, the findings suggest power generation across the hip and ankle is crucial for COD performance.

Finally, our review identified a range of kinematic and kinetic variables associated with COD performance. However, the influence of moderating factors, such as sex and training or playing experience, remains unclear. Notably, none of the included studies examined the role of playing or training experience in the biomechanical contributors to COD performance, reinforcing the need for future research in this area. Regarding sex differences, only one study included in our review [28] examined sex-specific kinetic and kinematic outcomes influencing COD performance across various COD tasks (180° 505 test, 90° T test and 45° V-cut). The authors reported that male basketball players demonstrated greater maximum velocity, acceleration, deceleration and centripetal force outputs during each COD task, leading to faster COD performance than female players across tests [28]. These findings indicated that lower limb joint-related biomechanical differences existed between sexes that may influence COD performance. Previously, female individuals were reported to exhibit greater knee abduction angles and moments than male individuals during 110° COD tasks [41] but COD performance was not examined. Subsequently, further research is warranted to confirm the influence of sex on the biomechanical determinants of COD performance, considering the variations in force application observed previously [28]. These sex-based variations in force application during high-speed tasks have been linked to differences in lower limb strength and neuromuscular characteristics [28, 42]. However, when strength is normalised to body mass (i.e. relative strength), these differences are substantially reduced or eliminated [43, 44]. Strength and the ability to generate high forces over short durations are critical determinants of athletic performance, particularly in tasks requiring rapid acceleration and deceleration such as COD tasks [43]. For example, strength-matched male and female pairs demonstrated similar force application when normalised to body mass, during an isometric mid-thigh pull task [43]. Therefore, future studies should also consider reporting relative strength of athletes to provide better insights into potential biomechanical differences between sexes [43–45] during COD tasks.

Our systematic review makes a unique and substantial contribution to practitioners’ current understanding of biomechanical contributors to COD performance (e.g., shorter GCT, greater approach and exit velocity, greater braking and propulsive forces). However, while our systematic review has yielded important findings, there are several limitations that should be considered. First, the influence of key moderating factors such as sex and training experience on COD performance [19, 22] was unclear from the current review. The majority (*n* = 10) of studies investigated COD performance in male athletes with possible biomechanical differences between sexes [19] likely to alter COD performance determinants for female athletes. Second, the training/playing experience of athletes was reported inconsistently across the studies, which limited our understanding of this factor on COD performance. For instance, novice athletes with limited experience may lack the muscular strength to effectively tolerate the forces during a COD task [12], or perform a COD task with poorer technique, potentially resulting in different biomechanical characteristics. Future research should consider training and playing experience when examining the biomechanical contributors to COD performance. Third, COD completion time was examined in this review, and future research should consider performance during different sub-phases of COD (i.e. acceleration, deceleration, turn and re-acceleration), particularly for sharper COD tasks where deceleration is more important. Last, the studies included in this review examined pre-planned COD tasks only. As movements within team sport settings involve unplanned actions, which are far more complex than pre-planned COD tasks [4, 46], future research should explore the biomechanical contributors to unplanned COD (also termed agility) for greater application among practitioners working in team sport settings. Unplanned COD tasks, common in team sport [47], may result in varied biomechanical contributors to performance compared to pre-planned COD tasks. For example, greater knee abduction moments were observed during an unplanned condition compared with pre-planned conditions [48]. Although these findings suggest there may be potential biomechanical distinctions between pre-planned and unplanned conditions of the same COD task, these differences have primarily been used to inform injury risk stratifications [20, 48, 49], with a limited focus on performance. In fact, the robust search conducted within this systematic review did not identify any studies comparing the biomechanical determinants of pre-planned and unplanned COD performances. Practitioners should consider these task-specific differences and the limitations of applying pre-planned COD training to unplanned scenarios [50]. This future work may provide valuable insights for athletes and coaches to target specific biomechanical attributes linked to quicker unplanned COD completion times and better prepare athletes for competitive environments (e.g. changing direction in response to a ball or an opponent).

## Training Recommendations

**Fig. 2:**
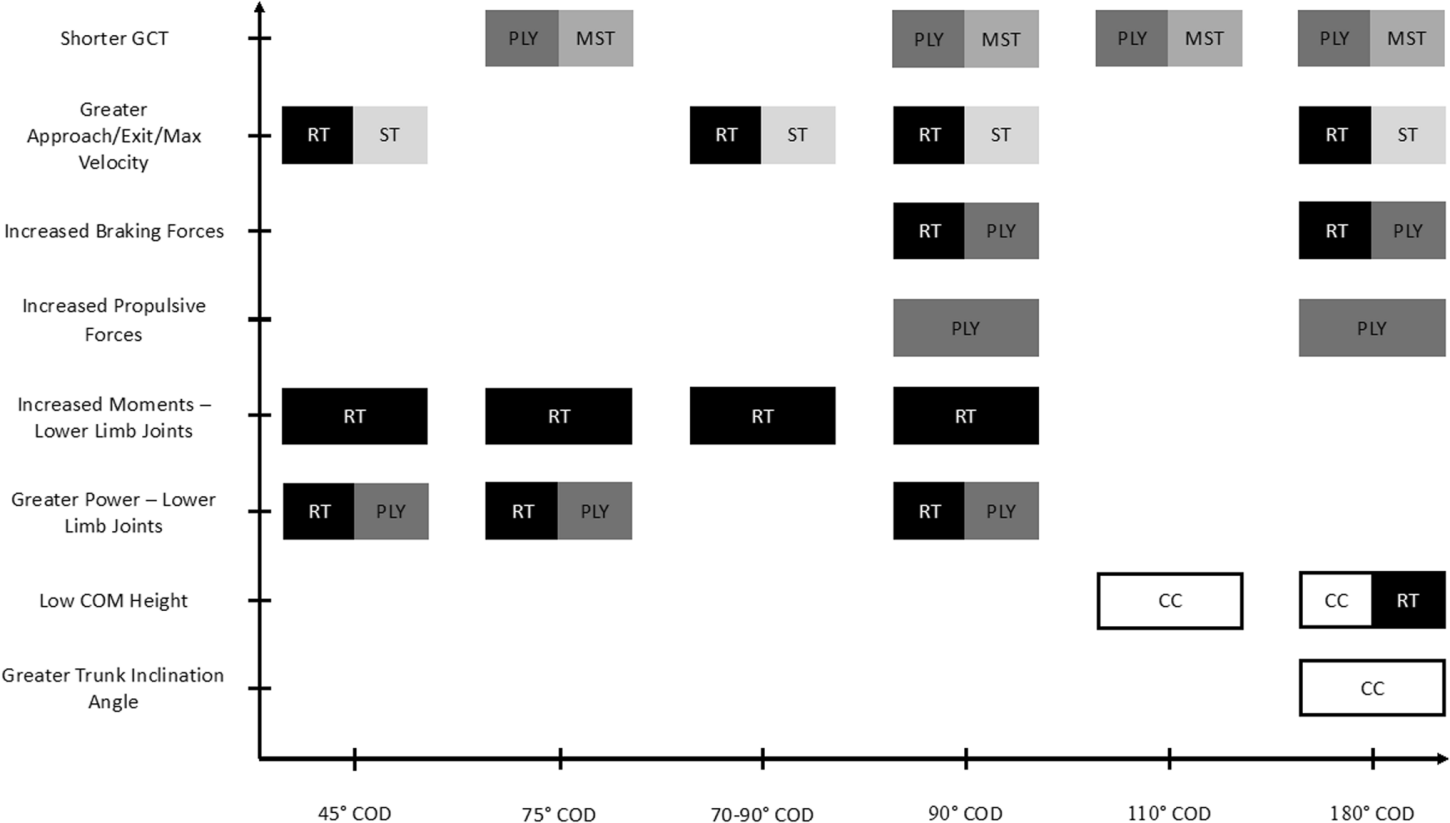
Training recommendations derived from prior studies. *CC* coaching cues, *COD* change of direction, *COM* centre of mass, *GCT* ground contact time, *PLY* plyometric training, *RT* resistance training, *Max* maximum, *MST* maximal strength training, *ST* sprint training

Our review identified important biomechanical determinants of COD performance that allowed the development of an elementary summary of training modalities for practitioners to enhance COD performance (Fig. 2). For example, plyometric exercises involving eccentric-concentric coupling [12] were recommended for most COD tasks based on prior studies linking shortened GCT with lower limb plyometric exercises [51–53]. Plyometric exercises have shown to influence physical qualities (e.g. pre-activation, reactive strength and explosive strength) [54, 55], which may contribute to reducing GCT. Further, practitioners can consider implementing maximal strength training, as this has been shown to enhance rapid force production during isometric tasks [56–58], which may help shorten GCT. Eccentric resistance training and horizontally oriented lower limb plyometric exercises [8, 59, 60] were recommended to address the greater braking and propulsive forces associated with greater COD performance (Fig. 2). It should be noted that increased velocity and braking forces during COD tasks can result in greater moments across the lower limb joints, which could have implications for injury risk, as greater sagittal plane knee moments have been linked to knee injuries [13, 29]. Subsequently, athletes should undertake relevant strength and conditioning training (e.g. eccentric strength of knee extensors) to aid effective deceleration, a crucial contributor to sharper COD cuts (≥ 90°) [16]. Additionally, plyometric exercises can aid in the development of peak power, exposure and tolerance to large moments across lower limb joints, shorter GCT and increased power output [17, 37, 61, 62]. These main training modalities in conjunction with sprint training to improve linear velocity [7] would be beneficial for improved COD performance (Fig. 2), although practitioners should consider the COD angle and the magnitude of linear velocity dependency as discussed earlier. For example, a sharper (≥ 90°) COD task may result in increased GCT [63] and reduced approach and exit velocity, as greater braking forces are required to decelerate and execute the desired COD [32]. Therefore, training should emphasise tolerating high knee joint loading, which may be developed through eccentric training such as flywheel inertial training [59] and accentuated eccentric exercises [64]. Additionally, minimising GCT and enhancing braking and propulsive forces can be targeted through plyometric training, which replicates the short contact durations observed in COD tasks and provides a stimulus for improving power output and joint moments [61], both of which contribute to COD performance. Importantly, a diverse range of plyometric exercises should be considered, as different movements provide varied mechanical stimuli across the hip, knee and ankle joints [61, 65, 66]. Notably, previous research suggests physically stronger athletes benefit more from plyometric training compared with weaker athletes [7]. Therefore, general strength training should not be overlooked, especially for weaker athletes, as it provides a solid foundation for the effective application of plyometric exercises. Finally, coaching cues for maintaining a low COM during the FFC for 110° and 180° COD tasks can result in improved COD performance, as a lower COM results in optimal position for braking and push-off as discussed previously [1, 18]. For instance, a coaching strategy (coaching cue, e.g. simultaneously bend hip, knee and ankle at the PFC) aimed at lowering the COM during a 180° COD would emphasise flexion of the hip, knee and ankle at PFC, as recommended by Dos Santos et al. [9]. Further, lower limb strength may also be important to enable greater lower extremity flexion and lowering of the COM as maximum squat strength was correlated with minimum COM height during a 180° COD [18]. Consequently, resistance training may be implemented to enhance lower extremity strength and facilitate COM positioning to enhance COD performance.

## Conclusions

Greater approach and exit velocities, shorter GCT, increased braking and propulsive forces, a greater trunk inclination angle, lower COM height, and increased moments and power at the hip, knee and ankle were confirmed as important biomechanical contributors to quicker COD performances. Coaches can use this information to prescribe effective training modalities for building physical fitness (e.g. rapid neuromuscular force generation through plyometrics and resistance training), and a skilled technique to optimise COD performance for sporting competition.

## Supplementary Information

Below is the link to the electronic supplementary material.

Supplementary file1 (DOCX 26 KB)

## Declarations

### Availability of data and material

All data and material included in this systematic review were retrieved from peer-reviewed publications with the relevant details provided in Tables 2, 3 and 4.

## References

[1] Welch N, Richter C, Franklyn-Miller A, Moran K. Principal component analysis of the biomechanical factors associated with performance during cutting. J Strength Cond Res. 2021;35(6):1715–23. 30664108 10.1519/JSC.0000000000003022

[2] Brughelli M, Cronin J, Levin G, Chaouachi A. Understanding change of direction ability in sport: a review of resistance training studies. Sports Med. 2008;38(12):1045–63. 19026020 10.2165/00007256-200838120-00007

[3] Young WB, Dawson B, Henry GJ. Agility and change-of-direction speed are independent skills: implications for training for agility in invasion sports. Int J Sports Sci Coach. 2015;10(1):159–69.

[4] Young W, Rayner R, Talpey S. It’s time to change direction on agility research: a call to action. Sports Med Open. 2021;7(1):12. 33580424 10.1186/s40798-021-00304-y PMC7881072

[5] Kozinc Ž, Sarabon N. Different change of direction tests assess different physical ability parameters: principal component analysis of nine change of direction tests. Int J Sports Sci Coach. 2021;17:1137–46.

[6] Dos’Santos T, Thomas C, Jones PA. The effect of angle on change of direction biomechanics: comparison and inter-task relationships. J Sports Sci. 2021;39(22):2618–31. 34278968 10.1080/02640414.2021.1948258

[7] Nygaard Falch H, Guldteig Raedergard H, van den Tillaar R. Effect of different physical training forms on change of direction ability: a systematic review and meta-analysis. Sports Med Open. 2019;5(1):53. 31858292 10.1186/s40798-019-0223-y PMC6923302

[8] Dos’Santos T, Thomas C, Jones PA. How early should you brake during a 180 degrees turn? A kinetic comparison of the antepenultimate, penultimate, and final foot contacts during a 505 change of direction speed test. J Sports Sci. 2021;39(4):395–405. 33377421 10.1080/02640414.2020.1823130

[9] Dos’Santos T, McBurnie A, Thomas C, Comfort P, Jones PA. Biomechanical determinants of the modified and traditional 505 change of direction speed test. J Strength Cond Res. 2020;34(5):1285–96. 31868815 10.1519/JSC.0000000000003439

[10] Dos’Santos T, Thomas C, Comfort P, Jones PA. Role of the penultimate foot contact during change of direction: implications on performance and risk of injury. Strength Cond J. 2019;41(1):87–104.

[11] Jones PA, Dos’Santos T, McMahon JJ, Graham-Smith P. Contribution of eccentric strength to cutting performance in female soccer players. J Strength Cond Res. 2022;36(2):525–33. 31800471 10.1519/JSC.0000000000003433

[12] Dos’Santos T, Thomas C, Comfort P, Jones PA. The effect of angle and velocity on change of direction biomechanics: an angle-velocity trade-off. Sports Med. 2018;48(10):2235–53. 30094799 10.1007/s40279-018-0968-3 PMC6132493

[13] Havens KL, Sigward SM. Cutting mechanics: relation to performance and anterior cruciate ligament injury risk. Med Sci Sports Exerc. 2015;47(4):818–24. 25102291 10.1249/MSS.0000000000000470

[14] Harper D, Cervantes C, Dyke M, Evans M, McBurnie A, Dos'Santos T, et al. The braking performance framework: practical recommendations and guidelines to enhance horizontal deceleration ability in multi-directional sports. Int J Strength Cond. 2024;4(1). 10.47206/ijsc.v4i1.351.

[15] Dos’Santos T, Thomas C, McBurnie A, Comfort P, Jones PA. Biomechanical determinants of performance and injury risk during cutting: a performance-injury conflict? Sports Med. 2021;51(9):1983–98. 33811615 10.1007/s40279-021-01448-3 PMC8363537

[16] Harper DJ, Jordan AR, Kiely J. Relationships between eccentric and concentric knee strength capacities and maximal linear deceleration ability in male academy soccer players. J Strength Cond Res. 2021;35(2):465–72. 29995690 10.1519/JSC.0000000000002739

[17] Marshall BM, Franklyn-Miller AD, King EA, Moran KA, Strike SC, Falvey ÉC. Biomechanical factors associated with time to complete a change of direction cutting maneuver. J Strength Cond Res. 2014;28(10):2845–51. 24662232 10.1519/JSC.0000000000000463

[18] Sasabe K, Sekine Y, Hirose N. The relationship between motor ability and change-of-direction kinematics in elite college basketball players. Int J Sport Health Sci. 2022;20:175–80.

[19] Thomas C, Dos’Santos T, Comfort P, Jones PA. Male and female soccer players exhibit different knee joint mechanics during pre-planned change of direction. Sports Biomech. 2024;23(1):118–31. 33115317 10.1080/14763141.2020.1830160

[20] Brown SR, Brughelli M, Hume PA. Knee mechanics during planned and unplanned sidestepping: a systematic review and meta-analysis. Sports Med. 2014;44(11):1573–88. 25015478 10.1007/s40279-014-0225-3

[21] DosʼSantos T, Thomas C, Jones PA, Comfort P. Mechanical determinants of faster change of direction speed performance in male athletes. J Strength Cond Res. 2017;31(3):696–705. 27379954 10.1519/JSC.0000000000001535

[22] Fox AS. Change-of-direction biomechanics: is what’s best for anterior cruciate ligament injury prevention also best for performance? Sports Med. 2018;48(8):1799–807. 29721837 10.1007/s40279-018-0931-3

[23] Page MJ, McKenzie JE, Bossuyt PM, Boutron I, Hoffmann TC, Mulrow CD, et al. The PRISMA 2020 statement: an updated guideline for reporting systematic reviews. BMJ. 2021;29(372): n71. 10.1136/bmj.n71 PMC8005924 33782057

[24] Ramachandran AK, Singh U, Connor JD, Doma K. Biomechanical and physical determinants of bowling speed in cricket: a novel approach to systematic review and meta-analysis of correlational data. Sports Biomech. 2024;23(3):347–69. 33428558 10.1080/14763141.2020.1858152

[25] Kmet LM, Cook LS, Lee RC. Standard quality assessment criteria for evaluating primary research papers from a variety of fields. Alberta Heritage Foundation for Medical Research (AHFMR)/HTA Report. 2004.

[26] Singh U, Ramachandran AK, Doma K, Connor JD. Exploring the influence of task and environmental constraints on batting and bowling performance in cricket: a systematic review. Int J Sports Sci Coach. 2023;18(6):2292–305.

[27] Jones PA, Thomas C, Dos’Santos T, McMahon JJ, Graham-Smith P. The role of eccentric strength in 180° turns in female soccer players. Sports (Basel). 2017;5(2):42. 29910402 10.3390/sports5020042 PMC5968983

[28] Baena-Raya A, Díez-Fernández DM, Martínez-Rubio C, Conceição F, López-Sagarra A. Kinetic and kinematic characteristics underpinning change of direction performance in basketball: a comparative study between sexes and tests. J Strength Cond Res. 2024;38(4):e182–8. 38300789 10.1519/JSC.0000000000004693

[29] McBurnie AJ, Dos’Santos T, Jones PA. Biomechanical associates of performance and knee joint loads during a 70–90° cutting maneuver in subelite soccer players. J Strength Cond Res. 2021;35(11):3190–8. 31268990 10.1519/JSC.0000000000003252

[30] Sasaki S, Nagano Y, Kaneko S, Sakurai T, Fukubayashi T. The relationship between performance and trunk movement during change of direction. J Sports Sci Med. 2011;10(1):112–8. 24149303 PMC3737904

[31] Suchomel TJ, Nimphius S, Stone MH. The importance of muscular strength in athletic performance. Sports Med. 2016;46(10):1419–49. 26838985 10.1007/s40279-016-0486-0

[32] Hader K, Palazzi D, Buchheit M. Change of direction speed in soccer: how much braking is enough? Kinesiology. 2015;47:67–74.

[33] Schreurs MJ, Benjaminse A, Lemmink K. Sharper angle, higher risk? The effect of cutting angle on knee mechanics in invasion sport athletes. J Biomech. 2017;3(63):144–50. 10.1016/j.jbiomech.2017.08.019 28886868

[34] Falch HN, Raedergard HG, van den Tillaar R. Effect of approach distance and change of direction angles upon step and joint kinematics, peak muscle activation, and change of direction performance. Front Sports Act Living. 2020;2: 594567. 33345172 10.3389/fspor.2020.594567 PMC7739774

[35] Havens KL, Sigward SM. Whole body mechanics differ among running and cutting maneuvers in skilled athletes. Gait Posture. 2015;42(3):240–5. 25149902 10.1016/j.gaitpost.2014.07.022

[36] Morin JB, Edouard P, Samozino P. Technical ability of force application as a determinant factor of sprint performance. Med Sci Sports Exerc. 2011;43(9):1680–8. 21364480 10.1249/MSS.0b013e318216ea37

[37] Kozinc Z, Smajla D, Sarabon N. The relationship between lower limb maximal and explosive strength and change of direction ability: comparison of basketball and tennis players, and long-distance runners. PLoS ONE. 2021;16(8): e0256347. 34407142 10.1371/journal.pone.0256347 PMC8372951

[38] Hewit J, Cronin J, Button C, Hume P. Understanding deceleration in sport. Strength Cond J. 2011;33:47–52.

[39] Noorkoiv M, Lavelle G, Theis N, Korff T, Kilbride C, Baltzopoulos V, et al. Predictors of walking efficiency in children with cerebral palsy: lower-body joint angles, moments, and power. Phys Ther. 2019;99(6):711–20. 31155663 10.1093/ptj/pzz041 PMC10468027

[40] Debaere S, Delecluse C, Aerenhouts D, Hagman F, Jonkers I. From block clearance to sprint running: characteristics underlying an effective transition. J Sports Sci. 2013;31(2):137–49. 22974278 10.1080/02640414.2012.722225

[41] Sigward SM, Cesar GM, Havens KL. Predictors of frontal plane knee moments during side-step cutting to 45 and 110 degrees in men and women: implications for anterior cruciate ligament injury. Clin J Sport Med. 2015;25(6):529–34. 25290102 10.1097/JSM.0000000000000155 PMC4387120

[42] Spiteri T, Hart NH, Nimphius S. Offensive and defensive agility: a sex comparison of lower body kinematics and ground reaction forces. J Appl Biomech. 2014;30(4):514–20. 24615296 10.1123/jab.2013-0259

[43] Comfort P, McMahon JJ, Lake JP, Ripley NJ, Triplett NT, Haff GG. Relative strength explains the differences in multi-joint rapid force production between sexes. PLoS ONE. 2024;19(2): e0296877. 38359039 10.1371/journal.pone.0296877 PMC10868802

[44] Nimphius S, McBride JM, Rice PE, Goodman-Capps CL, Capps CR. Comparison of quadriceps and hamstring muscle activity during an isometric squat between strength-matched men and women. J Sports Sci Med. 2019;18(1):101–8. 30787657 PMC6370970

[45] Nimphius S. Exercise and sport science failing by design in understanding female athletes. Int J Sports Physiol Perform. 2019;14(9):1157–8. 31553942 10.1123/ijspp.2019-0703

[46] Young W, Farrow D. A review of agility: practical applications for strength and conditioning. Strength Cond J. 2006;28:24–9.

[47] Singh U, Connor JD, Leicht AS, Brice SM, Doma K. Acute effects of prior conditioning activity on change of direction performance: a systematic review and meta-analysis. J Sports Sci. 2023;41(18):1701–17. 38124253 10.1080/02640414.2023.2293556

[48] Giesche F, Stief F, Groneberg DA, Wilke J. Effect of unplanned athletic movement on knee mechanics: a systematic review with multilevel meta-analysis. Br J Sports Med. 2021;55(23):1366–78. 34344709 10.1136/bjsports-2021-103933

[49] Almonroeder TG, Garcia E, Kurt M. The effects of anticipation on the mechanics of the knee during single-leg cutting tasks: a systematic review. Int J Sports Phys Ther. 2015;10(7):918–28. 26673276 PMC4675193

[50] Jones PA, Dos’Santos T. Multidirectional speed in sport: research to application. New York: Routledge; 2023.

[51] Asadi A, Arazi H, Young WB, de Villarreal ES. The effects of plyometric training on change-of-direction ability: a meta-analysis. Int J Sports Physiol Perform. 2016;11(5):563–73. 27139591 10.1123/ijspp.2015-0694

[52] McCormick BT, Hannon JC, Newton M, Shultz B, Detling N, Young WB. The effects of frontal- and sagittal-plane plyometrics on change-of-direction speed and power in adolescent female basketball players. Int J Sports Physiol Perform. 2016;11(1):102–7. 26023808 10.1123/ijspp.2015-0058

[53] Dello Iacono A, Martone D, Milic M, Padulo J. Vertical- vs. horizontal-oriented drop jump training: chronic effects on explosive performances of elite handball players. J Strength Cond Res. 2017;31(4):921–31. 27398920 10.1519/JSC.0000000000001555

[54] Ramírez-Campillo R, Andrade DC, Izquierdo M. Effects of plyometric training volume and training surface on explosive strength. J Strength Cond Res. 2013;27(10):2714–22. 23254550 10.1519/JSC.0b013e318280c9e9

[55] Ramirez-Campillo R, Thapa RK, Afonso J, Perez-Castilla A, Bishop C, Byrne PJ, et al. Effects of plyometric jump training on the reactive strength index in healthy individuals across the lifespan: a systematic review with meta-analysis. Sports Med. 2023;53(5):1029–53. 36906633 10.1007/s40279-023-01825-0 PMC10115703

[56] Aagaard P, Simonsen EB, Andersen JL, Magnusson P, Dyhre-Poulsen P. Increased rate of force development and neural drive of human skeletal muscle following resistance training. J Appl Physiol (1985). 2002;93(4):1318–26. 12235031 10.1152/japplphysiol.00283.2002

[57] Andersen JL, Aagaard P. Effects of strength training on muscle fiber types and size; consequences for athletes training for high-intensity sport. Scand J Med Sci Sports. 2010;20(Suppl. 2):32–8. 20840560 10.1111/j.1600-0838.2010.01196.x

[58] Andersen LL, Aagaard P. Influence of maximal muscle strength and intrinsic muscle contractile properties on contractile rate of force development. Eur J Appl Physiol. 2006;96(1):46–52. 16249918 10.1007/s00421-005-0070-z

[59] Bright TE, Handford MJ, Mundy P, Lake J, Theis N, Hughes JD. Building for the future: a systematic review of the effects of eccentric resistance training on measures of physical performance in youth athletes. Sports Med. 2023;53(6):1219–54. 37097414 10.1007/s40279-023-01843-y PMC10185653

[60] Singh J, Appleby BB, Lavender AP. Effect of plyometric training on speed and change of direction ability in elite field hockey players. Sports (Basel). 2018;6(4):144. 30424507 10.3390/sports6040144 PMC6315473

[61] Marshall BM, Moran KA. Which drop jump technique is most effective at enhancing countermovement jump ability, “countermovement” drop jump or “bounce” drop jump? J Sports Sci. 2013;31(12):1368–74. 23631690 10.1080/02640414.2013.789921

[62] Oliver JL, Ramachandran AK, Singh U, Ramirez-Campillo R, Lloyd RS. The effects of strength, plyometric and combined training on strength, power and speed characteristics in high-level, highly trained male youth soccer players: a systematic review and meta-analysis. Sports Med. 2024;54(3):623–43. 37897637 10.1007/s40279-023-01944-8 PMC10978689

[63] Condello G, Kernozek TW, Tessitore A, Foster C. Biomechanical analysis of a change-of-direction task in collegiate soccer players. Int J Sports Physiol Perform. 2016;11(1):96–101. 26024552 10.1123/ijspp.2014-0458

[64] Chaabene H, Prieske O, Negra Y, Granacher U. Change of direction speed: toward a strength training approach with accentuated eccentric muscle actions. Sports Med. 2018;48(8):1773–9. 29594958 10.1007/s40279-018-0907-3

[65] Van Lieshout KG, Anderson JG, Shelburne KB, Davidson BS. Intensity rankings of plyometric exercises using joint power absorption. Clin Biomech (Bristol). 2014;29(8):918–22. 25087112 10.1016/j.clinbiomech.2014.06.015

[66] Kotsifaki A, Korakakis V, Graham-Smith P, Sideris V, Whiteley R. Vertical and horizontal hop performance: contributions of the hip, knee, and ankle. Sports Health. 2021;13(2):128–35. 33560920 10.1177/1941738120976363 PMC8167345

