# Supplementary material for: Biomechanical Determinants of Change of Direction Performance: A Systematic Review
Source: Sports Med. 2025 Jul 16;55(9):2207–24. doi: 10.1007/s40279-025-02278-3 (PMC12476310; doi:10.1007/s40279-025-02278-3)
Supplement: Supplementary file 1 — Supplementary file1 (DOCX 26 KB) [file 40279_2025_2278_MOESM1_ESM.docx]

**Article title:** Biomechanical determinants of change of direction performance: a systematic review

**Journal name:** Sports Medicine.

**Author names:** Utkarsh Singh, Anthony S. Leicht, Jonathan D. Connor, Sara M. Brice, Adon Alves, Kenji Doma

**Affiliation and e-mail of the corresponding author:** Sport and Exercise Science, College of Healthcare Sciences, James Cook University, Townsville, Australia; Email: [utkarsh.singh@my.jcu.edu.au](mailto:utkarsh.singh@my.jcu.edu.au)

**Supplementary Table S1:** Measurement protocols for studies outcomes included in systematic review

| **Study** | **COD Type** | **COD Protocol** |
| --- | --- | --- |
| Baena-Raya et al. [28] | Modified 505 (180° COD), 90° COD and V cut (45° COD) | Modified 505: Athletes were instructed to run towards a line located 5 m from the starting point, where they were required to place either their left or right foot on the line. Subsequently, they were instructed to execute a 180° turn and sprint back through the finish line covering another 5 m.  Modified T test: Participants were instructed to sprint maximally towards a cone placed at a distance of 5m from the starting point and touch its base with their right hand. While facing forward and ensuring not to cross their feet, they shuffled to the left towards another cone positioned 2.5 m away and touched its base with their left hand. Subsequently, they shuffled 5 m towards the cone on the right and touched its base with their right hand. Then, they shuffled back 2.5 m to the left and touched its base. Finally, the players backpedaled 5 m to return to the starting point.  V cut: The participants executed a 25 m sprint, incorporating 4 COD at 45° every 5 m. |
| Dos'Santos et al. [21] | Modified 505 (180° COD) | Athletes were instructed to run towards a line located 5 m from the starting point, where they were required to place either their left or right foot on the line. Subsequently, they were instructed to execute a 180° turn and sprint back through the finish line covering another 5 m. |
| Dos'Santos et al. [9] | Modified and traditional 505 (180° COD) | Modified 505: Athletes were instructed to run towards a line located 5 m from the starting point, where they were required to place either their left or right foot on the line. Subsequently, they were instructed to execute a 180° turn and sprint back through the finish line covering another 5 m.  Traditional 505: Athletes were instructed to run towards a line located 15 m from the starting point, where they were required to place either their left or right foot on the line. Subsequently, they were instructed to execute a 180° turn and sprint back through the finish line covering another 5 m. |
| Dos'Santos et al. [15] | 90° COD | Cited previous studies. Participants were directed to sprint at their maximum effort for a distance of 15 m then perform a 90° COD on the force plate, and finally sprint for an additional 3 m. |
| Dos'Santos et al. [8] | Traditional 505 (180° COD) | Cited previous studies. Athletes were instructed to run towards a line located 15m from the starting point, where they were required to place either their left or right foot on the line. Subsequently, they were instructed to execute a 180° turn and sprint back through the finish line covering another 5m. |
| Havens and Sigward [13] | 45°and 90° | 45° COD: Participants were directed to sprint maximally for a distance of 7.5 meters, then plant their dominant foot and pivot away from it at a 45°, before continuing to run another 7.5 m.  90° COD: Participants were instructed to run maximally for a distance of 7.5 m, then plant their dominant foot and pivot away from it at a 90°, before continuing to run another 7.5 m. |
| Jones et al. [27] | Traditional 505 (180° COD) | Athletes were instructed to run towards a line located 15 m from the starting point, where they were required to place either their left or right foot on the line. Subsequently, they were instructed to execute a 180° turn and sprint back through the finish line covering another 5 m. |
| Jones et al. [11] | 70–90° COD | Participants were instructed to sprint through a series of timing gates placed 5 m from the midpoint of the final platform. Following this, they performed 70-90° cut upon making contact with the second force platform using their right leg. Subsequently, they proceeded through another set of timing gates positioned 3m away. Each participant commenced approximately 10 m behind the initial set of timing gates. |
| Marshall et al. [17] | 75° COD | Participants accelerated through a "start" gate, then proceeded maximally towards a cone positioned next to the force plate. After making a single complete foot contact on the force plate, they executed an approximately 75° cut before continuing to run maximally through a "finish" gate. |
| McBurnie et al. [29] | 70–90° COD | The initial timing gates were set 5 m from the center of the last force plate. Another pair of timing gates was placed 3 m away from the force plate at 70-90° to mark the completion point of the task. Participants were instructed to sprint maximally through the first set of timing gates. Upon reaching the first force plate (PFC), they were to plant their left foot, followed by their right foot on the final force plate (FFC). Immediately after, they were required to execute a 70-90° cut to the left and continue sprinting through the final set of timing gates.. |
| Sasabe et al. [18] | Modified 505 (180° COD ) | Athletes were instructed to run towards a line located 5 m from the starting point, where they were required to place either their left or right foot on the line. Subsequently, they were instructed to execute a 180° turn and sprint back through the finish line covering another 5 m. |
| Sasaki et al. [30] | Modified 505 (180° COD ) | Athletes were instructed to run towards a line located 5 m from the starting point, where they were required to place either their left or right foot on the line. Subsequently, they were instructed to execute a 180° turn and sprint back through the finish line covering another 5 m. |
| Welch et al. [1] | 45° and 110° COD | 45° COD: Participants were instructed to sprint maximally for a distance of 2 m, then plant their dominant foot and pivot away from it at a 45° before continuing to sprint for another 2 m.  110° COD: Not described. |

COD: change of direction; PFC: penultimate foot contact; FFC: final foot contact
